# Alcoholic Etiology, Severity of Liver Disease, and Post-Transplant Adherence Are Correlated with Worse Stanford Integrated Psychosocial Assessment for Transplantation (SIPAT) in Liver Transplant Candidates

**DOI:** 10.3390/jcm13133807

**Published:** 2024-06-28

**Authors:** Elisa Zanatta, Elisabetta Patron, Simone Messerotti Benvenuti, Filippo Pelizzaro, Francesco Paolo Russo, Martina Gambato, Giacomo Germani, Alberto Ferrarese, Alberto Zanetto, Federica Battermann, Francesca Buccheri, Chiara Cavalli, Rossana Schiavo, Marta Ghisi, Sara Pasquato, Paolo Feltracco, Umberto Cillo, Patrizia Burra, Marco Senzolo

**Affiliations:** 1 Multivisceral Transplant Unit-Gastroenterology, Department of Surgery, Oncology and Gastroenterology, Padua University Hospital, 35128 Padua, Italy; elisa.zanatta.2@studenti.unipd.it (E.Z.); francescopaolo.russo@unipd.it (F.P.R.); martina.gambato@aopd.veneto.it (M.G.); giacomo.germani@aopd.veneto.it (G.G.); alberto.ferrarese@unipd.it (A.F.); alberto.zanetto@unipd.it (A.Z.); marco.senzolo@aopd.veneto.it (M.S.); 2 Department of General Psychology, University of Padua, 35151 Padua, Italy; elisabetta.patron@unipd.it (E.P.); simone.messerotti@unipd.it (S.M.B.); marta.ghisi@aopd.veneto.it (M.G.); 3 Hospital Psychology Unit, Padua University Hospital, 35128 Padua, Italy; federica.battermann@unipd.it (F.B.); francesca.buccheri@aopd.veneto.it (F.B.); chiara.cavalli@aopd.veneto.it (C.C.); rossana.schiavo@aopd.veneto.it (R.S.); sara.pasquato@aopd.veneto.it (S.P.); 4 Padova Neuroscience Center (PNC), University of Padua, 35131 Padua, Italy; 5 Department of Surgery, Oncology and Gastroenterology, Padua University Hospital, 35128 Padua, Italy; filippo.pelizzaro@unipd.it; 6 Department of Medicine, UO Anesthesia and Intensive Care, University of Padua, 35128 Padua, Italy; paolo.feltracco@unipd.it; 7 Hepatobiliary Surgery and Liver Transplant Unit, Department of Surgery, Oncology and Gastroenterology, University of Padua, 35128 Padua, Italy; cillo@unipd.it

**Keywords:** liver transplantation, psychosocial assessment, Stanford Integrated Psychosocial Assessment for Transplantation score, overall outcomes, adherence to immunosuppressive medications

## Abstract

**Introduction**: Psychosocial pre-transplant evaluation in patients undergoing liver transplantation (LT) could help identify those patients at higher risk of pharmacological non-adherence, organ rejection, and mortality. The Stanford Integrated Psychosocial Assessment for Transplantation (SIPAT) is a validated tool for assessing LT candidates’ psychosocial well-being. Data on the ability of the SIPAT evaluation to predict post-transplant outcomes are sparse. **Material and Methods**: clinical and psychosocial data from a sample of 134 candidates for LT were analyzed. Moreover, the association between pre-transplant psychosocial evaluation and post-transplant clinical outcomes, including organ rejection, mortality, and immunosuppressant drug adherence, was calculated. **Results**: At the pre-transplant evaluation, patients who showed high SIPAT scores (77, 57%) also had more liver disease assessed by model for end-stage liver disease (MELD; F = 5.04; *p* < 0.05), alcoholic etiology (F = 35.80; *p* < 0.001), encephalopathy (F = 5.02; *p* < 0.05), and portal hypertension (F = 7.45; *p* < 0.01). Of the 51 transplant patients, those who had a high pre-transplant SIPAT score showed lower post-transplant immunosuppressive adherence, linked to more frequent immunological events. **Conclusions**: Patients with an alcoholic etiology of liver disease and more severe liver dysfunction are likelier to not adhere to medical prescriptions following transplantation. Current data suggests that this specific group of patients could benefit from early psychological pre-habilitation before undergoing liver transplantation.

## 1. Introduction

Liver transplantation has improved survival rates and quality of life for individuals experiencing end-stage liver disease. However, the demand for organ transplants significantly exceeds the available supply. Liver transplantation is an invasive procedure that, under optimal conditions, can lead to increased survival [1], better functional capacity [2], and improved quality of life [3]. Nevertheless, performing a multidisciplinary evaluation of biomedical, social, and psychological risk factors is essential for optimal performance. To streamline candidate selection and ensure optimal outcomes, liver transplant centers have incorporated psychosocial assessments as a routine part of their evaluations [4,5]. These assessments aim to measure psychosocial risk factors, including understanding the disease and transplant process, psychiatric history, support systems, compliance, and identifying strategies to mitigate the risk of unfavorable outcomes before transplantation [6].

Performing these assessments uniformly across different centers is challenging, as there are no standardized psychosocial evaluation guidelines for liver transplantation [7]. A pre-transplant screening tool, known as the Stanford Integrated Psychosocial Assessment for Transplantation (SIPAT) [4], was developed to enhance the identification of psychosocial risk factors. Initial studies have shown a significant association between SIPAT scores and overall outcomes after liver transplantation. Elevated scores are associated with post-transplant hospitalizations, organ rejection, failure of social support systems, and adverse psychiatric and psychosocial outcomes among all transplant recipients [8]. Furthermore, results have indicated that higher SIPAT scores are correlated with alcohol abuse relapses in transplant patients with alcohol-related liver disease [6].

Given that alcohol-related liver disease significantly impacts eligibility for the transplant list, and adherence to treatment and post-transplant follow-up plays a crucial role in maximizing the benefits of transplantation, the study aimed to establish correlations between the SIPAT assessment scores in patients awaiting liver transplants and the following factors: 1. etiology of the liver disease (specifically alcohol-related), 2. extent of liver damage, and 3. post-transplant therapeutic adherence. Specifically, it was hypothesized that higher SIPAT scores, reflecting poor psychosocial status, would be associated with an alcohol-related etiology of liver disease, a greater extent of liver damage, and lower post-transplant therapeutic adherence.

## 2. Materials and Methods

### 2.1. Participants

We conducted a single-center retrospective cohort study by analyzing the medical records of patients undergoing evaluation for liver transplantation at the Multivisceral Transplant Unit of the University of Padua from 1 January 2019 to 31 December 2022. The present study was conducted with the participants’ adequate understanding and written consent in accordance with the Declaration of Helsinki and was approved by the ethics committee (Protocol number: 18925).

The inclusion criteria were: (a) patients who underwent a clinical evaluation for liver transplantation (LT) between 1 January 2019 and 31 December 2022, and (b) patients who underwent a SIPAT evaluation.

Data from a subset of patients who underwent LT were analyzed. Specifically, this subset only included patients who underwent their first LT, were (c) transplant patients treated with immunosuppression, (d) patients with a follow-up of at least 1-year post-transplantation, (e) patients with at least 3 immunosuppressant determinations; (f) not combined transplants; and (g) first liver transplants.

Patients were excluded if they had: (a) received multiple transplants, (b) cognitive impairment, (c) insufficient data (no tacrolimus or everolimus level in the last year), and (d) died within 1 year after LT.

### 2.2. Protocol

All patients underwent thorough medical and psychosocial evaluations before LT between October 2019 and January 2022, with follow-up until 2023 at the Padova transplant center. Trained clinical psychologists performed psychosocial assessments and SIPAT administration. After the initial evaluation, the multidisciplinary committee determined the transplant candidate based on the data from the multidisciplinary evaluation, including transplant hepatology, transplant surgery, psychosocial symptoms, and registered dietitians, on a case-by-case basis. For patients who underwent LT, routine post-transplant follow-up included evaluations at 1, 3, 6, 9, 12, 18, 21, and 24 months after LT. Protocols were stable throughout the study period.

### 2.3. Clinical and Psychosocial Variables

The following sociodemographic, clinical, and psychosocial information was collected for each patient during evaluation for liver transplantation: 1. age at transplantation (years), 2. sex (female/male), 3. education (years), 4. alcohol-related liver disease (present/absent), 5. Model for End-Stage Liver Disease (MELD) score, 6. clinically significant portal hypertension (present/absent), 7. encephalopathy (present/absent), 8. Charlson Comorbidity Index (CCI), 9. Internal Normalized Ratio (INR), 10. bilirubin, 11. creatinine, and 12. Stanford Integrated Psychosocial Assessment for Transplantation (SIPAT) score. 

The SIPAT is a multifaceted, semi-structured tool for psychosocial evaluation in solid organ transplants. The Italian version of SIPAT has shown excellent psychometric characteristics, including high inter-rater reliability and predictability of listing outcomes [9]. It is composed of 18 items grouped into 4 psychosocial factors: (1) patient’s readiness and illness management (score range = 0–24), (2) social support system (score range = 0–20), (3) psychological stability and psychopathology (score range = 0–37), and (4) lifestyle and effect of substance use (score range = 0–29). The sum of each item’s scores provides a total SIPAT score (total score range = 0–110); scoring categories are excellent candidate (total score ranging from 0 to 6), good candidate (total score ranging from 7 to 20), minimally acceptable candidate (total score ranging from 21 to 39), poor candidate (total score ranging from 40 to 69), and high-risk candidate (total score > 70). SIPAT was included in the psychosocial assessments for LT; however, no absolute thresholds were considered acceptable or unacceptable for transplant waitlisting because each candidate was considered individually.

In the subset of patients who underwent LT, the following information was collected: 1. acute liver rejection (considered within 3 months post-transplant) from histological diagnosis (present/absent); 2. chronic liver rejection from histological diagnosis (present/absent), de novo immune-mediated hepatitis; 3. tacrolimus blood levels at 1, 3, 6, 9, 12, 18, 21, 24 months; 4. CCI; 5. recurrence of alcohol abuse (present/absent); 6. post-transplantation mortality (present/absent). 

Adherence to pharmacological treatment was calculated as indices of intra-patient variability (IPV) over nine assessments two years after transplantation using the medication level variability index (MLVI) of serum levels of tacrolimus and everolimus. The MLVI calculates the degree of fluctuation of medication blood levels in individual patients over time by determining the patient’s target range and standard deviation of 3 or more blood levels. A higher MLVI reflects more significant fluctuations, meaning the patient had more erratic pharmacological adherence. As per previous data, MLVI can also be regarded as a clinically meaningful dichotomous variable, with a value exceeding two units indicating clinically significant non-adherence [10].

### 2.4. Statistical Analyses

Missing data were handled by case exclusion. Patients were categorized into two groups based on a SIPAT score < 21 (i.e., excellent or good candidate) or a SIPAT score ≥ 21 (i.e., minimally acceptable or poor transplant candidate), creating a binary total SIPAT score variable. It should be noted that no patient included in the present study showed a SIPAT total score > 70, meaning they were a high-risk candidate. A SIPAT score greater than 21 has been associated with lower pharmacological adherence and a higher risk of allograft rejection [11]. The MELD score was logarithmically transformed to normalize the distribution. Regarding the subset of patients who underwent LT, acute and chronic liver rejection were evaluated based on the histological diagnosis. Since the number of patients with chronic liver rejection was small, we decided to merge the data from acute (13 patients, 25%) and chronic (4 patients, 8%) liver rejection. Baseline characteristics between patients with low risk (SIPAT score < 21) and high risk (SIPAT score ≥ 21) were compared using *t*-tests and chi-squared tests, as appropriate. Descriptive statistics were used to summarize sample characteristics (see Table 1).

Multivariate regression was applied to calculate the association between the groups (low risk and high risk) based on the total SIPAT score, lnMELD, liver disease etiology (alcoholic versus non-alcoholic), encephalopathy (present/absent), and portal hypertension (present/absent) that were included as dependent variables while controlling for age and sex.

In the subset of patients who underwent LT, to evaluate the role of the SIPAT score in predicting adherence to pharmacological therapy, logistic regression was calculated with the groups (low risk and high risk) based on the total SIPAT score as the independent variable and tacrolimus MLVI as the dependent variable (the same model was not applied to everolimus MLVI due to the smaller sample size for these data). Furthermore, logistic regression models were applied to evaluate the role of the SIPAT score in predicting organ rejection (including acute and chronic rejection) and the mortality rate after LT.

Finally, since tacrolimus MLVI is the most effective predictor for patients at higher risk of rejection [12], to evaluate the association between adherence to pharmacological therapy and the risk of immunological events after LT, logistic regression was calculated with tacrolimus MLVI as the independent variable and rejection (present or absent) as the dependent variable.

A *p*-value < 0.05 was considered statistically significant. All of the statistical calculations were performed using SPSS (version: 29.0.1.0, Chicago, IL, USA).

## 3. Results

### 3.1. Characteristics of Included Patients at Pre-Transplant Evaluation

**Figure 1:**
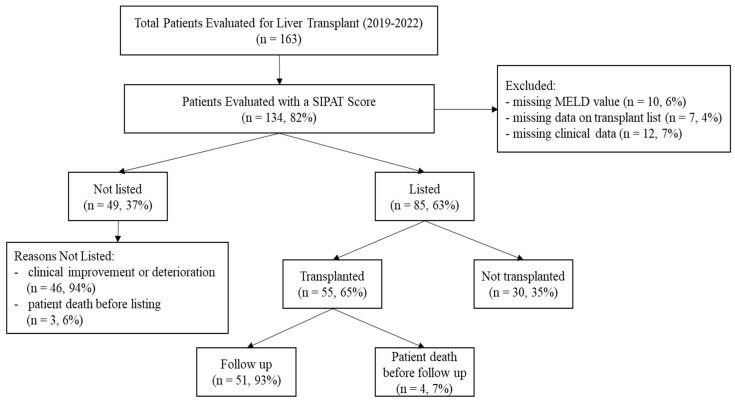
CONSORT diagram of patient enrollment.

A total of 163 patients were initially enrolled; successively, 10 (6%) patients were excluded due to missing MELD values, 7 (4%) patients were excluded due to missing data from the clinical transplant list, and 12 (7%) patients were excluded due to missing clinical data (e.g., INR, bilirubin, creatinine, clinically significant portal hypertension, encephalopathy). Consequently, the analyses were conducted on 134 (82%) patients (see Figure 1). The mean age at evaluation was 57.80 ± 9.49 years (range 28–71 years), and 88 (66%) were male. On average, the level of education was 10.92 (2.21 years) (range 5–18 years). The whole group had a lnMELD score of 1.10 ± 0.18 (the raw mean MELD score was 13.9 ± 6.28; range 6–30); ALD was present in 67 patients (50%), and all these patients reported abstinence for longer than six months. Clinically significant portal hypertension was observed in 104 (78%), and encephalopathy was diagnosed in 30 patients (22%). The mean CCI was 5.4 ± 2.0 (range 1–10; see Table 1).

The average total SIPAT score was 23.93 ± 12.99 (range 0–63): seventy-seven (57%) patients were identified as high-risk, and 57 (43%) as low-risk.

### 3.2. Association between Pre-Transplant Clinical Characteristics and SIPAT

Comparison through the multivariate regression (see Table 2) showed that patients with high risk based on the total SIPAT score showed a higher level of lnMELD (F = 5.040; *p* = 0.026), more often they were diagnosed with ALD (F = 35.801; *p* < 0.001), more often they showed encephalopathy (F = 5.023; *p* = 0.027), and clinically significant portal hypertension (F = 7.455; *p* = 0.007).

Additionally, the percentage of transplant patients was compared according to the SIPAT score for ALD and non-ALD etiologies. In the group of patients with ALD (see Table 3), 13 were classified as good candidates based on the SIPAT score, while 54 (81%) were identified as high-risk SIPAT candidates (35 minimally acceptable candidates and 19 poor candidates). Transplantations were performed for 10 (77%), 14 (40%), and 3 (16%) patients with good, minimally acceptable, and poor SIPAT scores, respectively (chi-square: 11.994, *p* = 0.0005).

Similarly, in the group of patients without ALD (see Table 4), 44 were classified as good candidates based on the SIPAT score, while 23 (34%) were identified as high-risk SIPAT candidates (18 minimally acceptable candidates and 5 poor candidates). Transplantations were performed for 20 (45%), 7 (39%), and 1 (20%) patients with good, minimally acceptable, and poor SIPAT scores, respectively. The Freeman–Halton extension of Fisher’s exact test was used for the analyses of the patients without ALD (*p* = 0.574). An accurate *p*-value could not be estimated using the chi-squared test due to the insufficient sample size in more than one cell of the table.

### 3.3. Association between Adherence to Immunosuppressive Therapy or Organ Rejection and SIPAT

Of the 134 patients examined and assessed for LT, 85 (63%) were actively listed for transplantation. Three patients (2%) died before listing, and 46 (34%) were excluded due to clinical improvement or deterioration. Among the 85 patients on the transplant list, 55 (65%) underwent LT; nine patients (10%) died before transplantation, and 21 (25%) were not transplanted at the time of the study. Of the 55 patients who underwent LT, 4 (7%) died during the surgical intervention or immediately after; therefore, they were excluded from further analyses. The final analysis of post-transplant patients included 51 patients (91%).

The mean age of patients undergoing liver transplantation was 56.96 ± 10.02 years, and 38 (75%) were male. lnMELD was 1.12 ± 0.18 (raw MELD score was 14.33 ± 6.18) at transplant; post-transplantation CCI was 5.57 ± 2.0. The median follow-up was 24 months. Alcohol recurrence was 3.7% among the 27 transplant patients for exotoxic etiology (1 patient out of 27). Acute rejection was identified in 13 (25%) patients, while chronic rejection was identified in four subjects (7%).

Adherence to pharmacological treatment was calculated as the MLVI of tacrolimus serum level in 51 (100%) of the post-transplant patients and as the MLVI of everolimus serum level in 24 (47%) of the post-transplant patients. Pharmacological treatment adherence evaluated using MLVI at scheduled intervals revealed a tacrolimus MLVI of 1.60 ± 0.77 (range 0.31–4.10) and an everolimus MLVI of 1.24 ± 0.78 (range 0.28–3.87). The sample of post-transplant patients was categorized according to adherence to pharmacological therapy using a median split on tacrolimus MLVI (tacrolimus MLVI median = 1.47). A logistic regression was then calculated to evaluate the role of the SIPAT category (low risk and high risk) in predicting the category of adherence to pharmacological treatment, controlling for age. The results showed that high-risk SIPAT patients were more than three times more likely than the low-risk group to have an MLVI higher than the median of the whole group (β = −1.221, OR = 3.392; CI = 1.07–10.26, *p* = 0.038, see Table 5).

Regarding clinical outcomes, no association was found between the SIPAT category (low risk and high risk) and organ rejection (including acute and chronic rejection; β = −0.012, OR = 0.988; CI = 0.32–3.04, *p* = 0.983) or mortality peri-transplant or post-transplant mortality (β = −0.486, OR = 0.615; CI = 0.09–4.01, *p* = 0.611).

Finally, the logistic regression in post-transplant patients evaluating the relationship between adherence to immunosuppressive therapy (good adherence MLVI < 2; scarce adherence MLVI ≥ 2) showed no statistically significant results (β = −0.993, OR = 2.700; CI = 0.75–9.57, *p* = 0.127, see Table 6). However, it should be noted that half of the patients with limited adherence (7/14; 50%) experienced immunological events compared to less than a third of patients with good adherence (10/37; 27%) who showed clinical signs of organ rejection.

## 4. Discussion

The primary objective of this study was to evaluate the association between pre-transplant psychosocial assessment conducted through the SIPAT and clinical characteristics that are well-known risk factors associated with worse outcomes after LT. Furthermore, this study aimed to evaluate how pre-transplant psychosocial evaluation predicted post-transplant adherence to pharmacological therapy, as well as organ rejection and mortality risk. Results highlighted that patients with a high pre-transplant SIPAT score had a higher MELD score and were more likely to have alcohol-related liver disease [13], encephalopathy, and clinically significant portal hypertension. The present results add to the findings reported by Deutsch-Link et al. [11] and Daniel et al. [14], who showed that pre-transplant patients with high psychosocial risk (SIPAT score ≥ 21) are more likely to have ALD etiology and a higher MELD score. Moreover, the present results showed that a high SIPAT score was associated with other relevant clinical factors, such as the presence of encephalopathy and clinically significant portal hypertension, in patients undergoing evaluation for LT. No other studies in the literature have documented this particular correlation.

Regarding the ability of the SIPAT score to predict a clinically relevant variable in post-transplant patients, statistical analysis revealed that patients in the high-risk SIPAT category were more than three times more likely to have lower pharmacological treatment adherence (that is, higher tacrolimus MLVI) compared with patients in the low-risk SIPAT category, indicating the potential of pre-transplant SIPAT evaluation in predicting post-transplant pharmacological treatment adherence.

In line with the present results, Deutsch-Link et al. [11] reported that a high pre-transplant SIPAT score was associated with post-transplant alcoholic relapse and low adherence to immunosuppression [15], measured by the tacrolimus coefficient of variation. In the present study, the analysis of the relationship between post-transplant pharmacological adherence (tacrolimus MLVI) and organ rejection did not produce a statistically significant result. However, it hinted at a potential trend in the non-adherence group. A statistically significant relationship could be absent due to the low number of organ rejection or mortality cases.

Among the SIPAT subclasses, only Deutsch-Link et al. [11] found a correlation between subclass 1 (patient’s readiness level) and the risk of post-transplant rejection at three months, while other authors such as Monaldo et al. [8] did not highlight any correlation with graft failure or rejection. Lindsay et al. [16] have reported that SIPAT and MELD scores were predictive of rejection; however, this association did not withstand multivariable analysis. Additionally, they did not find any correlation between SIPAT scores and mortality.

In our series, patients with SIPAT > 21, compared to those with SIPAT < 21, exhibited significantly higher scores in subclasses 1 and 4 relative to. However, SIPAT and its subdomains are not reliable in alcohol relapse models [13], and we found no correlations with relapse due to the low number of events (1/27).

SIPAT stands as one of several psychosocial assessment tools employed in evaluating patients with end-stage diseases, aiding in comprehending their overall health status and assessing their suitability for organ transplantation [14,17].

Thode et al. [18] highlighted how the different psychosocial tools are somewhat imperfect in predicting medical outcomes (mortality, rejection, organ failure) in the post-transplant compared to predicting psychosocial outcomes (non-adherence, alcohol relapse, quality of life). The imperfection may probably arise because the correlation between the psychosocial tool scores and the outcomes is made only in the group of transplant patients and not with the entire population subjected to a pre-transplant test. Furthermore, the follow-up period could also influence these different performances of the psychosocial tools: with too short follow-ups (for example, one year), one could miss events that appear after 3–4 years.

It should be noted that caution must be taken when drawing inferences about the association between the SIPAT score and the severity of liver disease. In the present study, patients with ALD presented a higher SIPAT score (mean SIPAT score ± SD = 29.66 ± 13.54) compared to those without ALD (mean SIPAT score ± SD = 18.21 ± 9.50; t = −5.67, *p* < 0.001), in line with data reported in the literature [14]. Therefore, it is likely that the presence of ALD is often present in the cooccurrence of alcohol use disorder, which in turn aggravates encephalopathy and portal hypertension and increases the risk of a higher SIPAT score. This, in turn, could be associated with a higher risk of post-transplant relapse and low post-transplant adherence.

Data about the correlation between SIPAT and recidivism in alcohol consumption after liver transplantation are limited by the low number of patients and the intrinsic selection bias during lower transplants amongst poor candidates according to SIPAT scores.

Our study had several limitations. This was a single-center, observational cohort study with the potential for unmeasured confounding variables and limited generalizability to other transplant programs. Although some patients had missing SIPAT assessments, we compared demographic and clinical characteristics between those with complete and missing data.

The success of a transplant depends on various aspects of candidate selection, encompassing not only clinical factors but also psychosocial elements. This is particularly crucial for long-term outcomes and the success of candidates with alcohol-related issues [19]. With a shift in candidate selection prioritizing survival benefits over strict adherence to the 6-month abstinence rule, it becomes increasingly important to identify objective parameters that can flag individuals at risk of poor outcomes due to non-adherence, which can sometimes be subtle and not manifest as a clear decision to take or refrain from medication. The present results suggest that pre-transplant psychosocial evaluation could help physicians recognize patients with a potential risk of non-adherence, and it could also lead to the design of new tools to better understand non-adherence after LT and targeted interventions to promote LT patients’ adherence. It also highlights the complexity of these factors and the need for continued research to refine risk-assessment tools and optimize patient management strategies in liver transplantation.

## 5. Conclusions

Pre-transplant psychosocial assessment via SIPAT offers valuable insights into liver transplant candidates’ clinical profiles and post-operative outcomes. Pre-transplant elevated SIPAT scores hold the potential to predict medication adherence post-transplant and a higher risk of immunological events. These data suggest that more severe patients with alcoholic liver disease could benefit from early psychological pre-habilitation before undergoing liver transplantation.

## Figures and Tables

**Table 1:** Characteristics of included patients at pre-transplant evaluation.

	N = 134
Age, years (m, ±SD)	57.80 ± 9.49
Sex, male (N, %)	88 (66)
Education, years (m, ±SD)	10.92 ± 2.21
ALD (Yes, %)	67 (50)
Encephalopathy (Yes, %)	30 (22)
Portal hypertension (Yes, %)	104 (78)
MELD (m, ±SD)	13.90 ± 6.28
lnMELD (m, ±SD)	1.10 ± 0.18
Bilirubin (mg/dL) (m, ±SD)	3.16 ± 4.63
Creatinin (mg/dL) (m, ±SD)	1.0 ± 0.67
SIPAT total score (m, ±SD)	23.93 ± 12.99
Patient’s readiness level	8.56 ± 4.59
Social support system	4.81 ± 2.89
Psychological suitability and psychopathology	4.78 ± 3.81
Lifestyle and effect of substance use	5.78 ± 5.29
SIPAT, high risk (N, %)	77 (57)

Note: ALD = alcohol-related liver disease; MELD = Model for End-Stage Liver Disease; lnMELD = natural logarithm of the Model for End-Stage Liver Disease; SIPAT = Stanford Integrated Psychosocial Assessment for Transplantation.

**Table 2:** Multivariate regression assessed the association between the SIPAT groups (high and low risk) and clinically relevant variables.

	High Risk (N = 77)	Low Risk (N = 57)	F	η_p_^2^	Observed Power	*p*
Age, years (m, ± SD)	58.04 ± 7.27	57.47 ± 11.91	0.103	0.001	0.062	0.749
Sex, male (N, %)	55 (71)	33 (58)	2.945	0.022	0.399	0.088
lnMELD (m, ± SD)	1.13 ± 0.17	1.07 ± 0.19	5.040	0.037	0.606	0.026
ALD (N, %)	54 (70)	13 (23)	35.801	0.215	1.000	<0.001
Encephalopathy (N, %)	22 (29)	7 (12)	5.023	0.037	0.604	0.027
Portal hypertension (N, %)	66 (86)	37 (66)	7.455	0.054	0.773	0.007

Note: ALD = alcohol-related liver disease; lnMELD = natural logarithm of the Model for End-Stage Liver Disease; SIPAT = Stanford Integrated Psychosocial Assessment for Transplantation.

**Table 3:** Patients with ALD stratified according to the SIPAT score.

	SIPAT Score Good Candidate (n = 13)	SIPAT Score Minimally Acceptable Candidate (n = 35)	SIPAT Score Poor Candidate (n = 19)
Not Transplanted	3 (23%)	21 (60%)	16 (84%)
Transplanted	10 (77%)	14 (40%)	3 (16%)

Chi-square test: 11.994; *p* = 0.0005. Note: ALD = alcohol-related liver disease; SIPAT = Stanford Integrated Psychosocial Assessment for Transplantation.

**Table 4:** Patients without ALD stratified according to the SIPAT score.

	SIPAT Score Good Candidate (n = 44)	SIPAT Score Minimally Acceptable Candidate (n = 18)	SIPAT Score Poor Candidate (n = 5)
Not Transplanted	24 (55%)	11 (61%)	4 (80%)
Transplanted	20 (45%)	7 (39%)	1 (20%)

Freeman–Halton extension of the Fischer’s exact test *p* = 0.574. Note: ALD = alcohol-related liver disease; SIPAT = Stanford Integrated Psychosocial Assessment for Transplantation.

**Table 5:** Characteristics of transplant patients.

	N = 51
Age, years (m, ± SD)	56.96 ± 10.02
Sex, Male (N, %)	38 (75)
ALD (Yes, %)	27 (53)
lnMELD (m, ± SD)	1.12 ± 0.18
Acute rejection	13 (25)
Chronic rejection	4 (7)
Mean Tacrolimus MLVI (m, ± SD)	1.6 ± 0.77
Median Tacrolimus MLVI	1.47
SIPAT high risk group	27 (53)

Note: ALD = alcohol-related liver disease; lnMELD = natural logarithm of the Model for End-Stage Liver Disease; SIPAT = Stanford Integrated Psychosocial Assessment for Transplantation; MLVI = medication level variability index.

**Table 6:** Outcomes in transplant patients according to SIPAT risk group.

	Adherence (MLVI > 2)	Organ Rejection	Mortality
SIPAT high risk group	6 (22)	10 (35.7)	2 (7.1)
SIPAT low risk group	3 (12.5)	9 (36)	3 (11.1)
Statistic	*p* = 0.13	*p* = 0.98	*p* = 0.61

Note: SIPAT = Stanford Integrated Psychosocial Assessment for Transplantation; MLVI = medication level variability index.

## Data Availability

Data is unavailable due to privacy or ethical restrictions.
